# Prevalence and characteristics of resistant hypertension at primary clinics in Korea: a nationwide cross-sectional study

**DOI:** 10.1186/s40885-016-0043-z

**Published:** 2016-01-31

**Authors:** Kwang No Lee, Jin Oh Na, Cheol Ung Choi, Hong Euy Lim, Jin Won Kim, Eung Ju Kim, Seung-Woon Rha, Hong Seog Seo, Dong Joo Oh, Chang Gyu Park

**Affiliations:** 1 Department of Cardiology, Korea University Anam Hospital, Seoul, Korea; 2 Department of Cardiology, Korea University Guro Hospital, Seoul, Korea

**Keywords:** Resistant hypertension, Prevalence, Primary clinics

## Abstract

**Background:**

Although resistant hypertension (RH) is known to be associated with higher rates of cardiovascular events than is non-RH, there are no reported data on the prevalence of RH in Korean patients. We evaluated the prevalence and characteristics of RH among hypertensive patients treated at primary clinics in Korea.

**Methods:**

Between August 2010 and January 2011, 247 primary care physicians enrolled 3088 patients with essential hypertension. We acquired demographic and anthropometric data using a questionnaire, evaluated blood pressure, and conducted a variety of laboratory tests using serum and urine. RH was defined as systolic blood pressure ≥140 mmHg or diastolic blood pressure ≥90 mmHg with the use of three antihypertensive agents of different classes, including a diuretic, or controlled hypertension with the use of four or more medications.

**Results:**

We analyzed 3088 patients with hypertension, 48.3 % of whom were men. The mean age of patients was 64.3 ± 11.3 years and the prevalence of RH was 7.9 %. Patients with RH were more likely to be men, and to have higher waist circumference, increased blood levels of HbA1c, triglycerides, and serum creatinine, lower blood levels of high-density lipoprotein (HDL), and higher rates of current smoker, history of heart failure or coronary artery disease, and electrocardiographic left ventricular hypertrophy (LVH), than were patients with non-RH (all comparisons, *P* < 0.05). In the multivariate analysis, RH was shown to be significantly associated with the following conditions: presence of electrocardiographic LVH (odds ratio [OR] 2.23, 95 % confidence interval [CI] 1.34–3.71), current smoker (OR 1.75, 95 % CI 1.27–2.40), renal impairment (OR 1.65, 95 % CI 1.23–2.22), abdominal obesity (OR 1.60, 95 % CI 1.20–2.13), and cardiovascular diseases (OR 1.50, 95 % CI 1.04–2.17).

**Conclusions:**

The prevalence of RH was relatively low at primary clinics in Korea compared with the prevalence reported in other countries. RH was associated with electrocardiographically confirmed LVH, renal impairment, current smoker, abdominal obesity, and cardiovascular diseases. These are the first reported data of RH in Korea. Our findings may be helpful in the early detection and thorough clinical management of patients with RH at primary clinics.

## Background

Resistant hypertension (RH) was defined by the seventh Joint National Committee as the failure to achieve a target blood pressure despite maintaining full doses of three antihypertensive agents of different classes, one of which should be a diuretic [1]. Following a 2008 statement by the American Heart Association, controlled hypertension using at least four medications is also considered as RH [2].

Although the specific prognostic implications of RH have not been well evaluated, RH is frequently associated with cardiovascular risk factors, such as older age, diabetes mellitus, obesity, obstructive sleep apnea, left ventricular hypertrophy (LVH), and chronic kidney disease [2, 3]. Among patients in a retrospective cohort study, those with RH had an higher rate of cardiovascular events, as shown by an adjusted hazard ratio of 1.47 (95 % confidence interval [CI], 1.33–1.62; *P* < 0.001), over the mean follow-up period of 3.8 years than did those with non-RH [4]. Therefore, precise statistical data of RH and aggressive treatment of this condition may be necessary to reduce cardiovascular events.

However, to our knowledge, the exact prevalence of RH in Korea has yet to be reported. Patients who have difficulty controlling their blood pressure or who are suspected to have RH are usually referred from primary clinics to the hospital, where cardiologists who specialize in hypertension manage their care. Therefore, the investigation of the clinical management of RH in primary clinics may be insufficient to capture the prevalence of this disease, even though the prevalence of RH is reportedly 11 ~ 21 % at tertiary facilities and 10 % at primary clinics in the United States [5, 6].

Hypertension is often present as part of the metabolic syndrome and is associated with insulin resistance. Higher rates of diabetes mellitus or obesity may be present in patients with RH compared with those who have non-RH. Accurate information regarding the prevalence and outcome of RH is important for managing and improving the prognosis of this condition. Identifying and targeting high-risk patients with hypertension can increase the cost effectiveness of primary clinics. Therefore, we investigated the prevalence of RH and evaluated the characteristics of patients with RH in primary clinics in Korea.

## Methods

### Study population

Patients who were older than 18 years with essential hypertension and who visited primary clinics in 2010 were eligible to participate in this cross-sectional study. Patients were excluded if they had the following conditions: secondary hypertension, white-coat hypertension, acute myocardial infarction, unstable angina, acute phase of stroke, peripheral artery disease, or uncontrolled diabetes mellitus (HbA1c > 9.0 %). Two hundred and forty-seven physicians in 230 primary clinics participated in this study through the network that was organized in a previous investigation [7]. The primary clinic was chosen randomly according to the proportion of the population in each city or province. This study was approved by the Institutional Review Board of the Korea University Guro Hospital.

Physicians measured blood pressure using an electronic sphygmomanometer (OMRON MX-3, Omron Healthcare, Kyoto, Japan). After the patient had been seated quietly for 5 min, blood pressure was measured with the right arm of the patient supported at the level of the heart. We obtained two or three blood pressure measurements, 30 s apart. If blood pressure was measured twice, the average value was assigned to the blood pressure of the patient. If it was measured three times, the second and the third values were used to calculate the average of systolic and diastolic blood pressure.

Each patient enrolled in the study was given a self-administered questionnaire in order to collect data on demography, lifestyle, and a family history of hypertension and cardiovascular disease. The physicians also recorded anthropometrical parameters, such as comorbidities and antihypertensive medications.

An 8-h fasting blood sample was collected for the measurement of hemoglobin A1c, fasting plasma glucose, and blood lipids. Urinary analysis was also performed to measure albumin concentration in the spot urine and the albumin/creatinine ratio was estimated to compensate for variations in urinary concentration.

The body mass index (BMI) was calculated from the patient’s height and weight. The waist circumference was measured at the narrowest part of the waist between the lowest rib and the iliac crest.

### Definitions

The Eighth Joint National Committee defined hypertension as either office systolic blood pressure level of more than 140 mmHg or diastolic blood pressure of less than 90 mmHg [8]. RH was defined as the failure to reach target blood pressure despite full doses of an appropriate three antihypertensive medications of different classes, including a diuretic, or controlled hypertension that required 4 or more medications [2].

Abdominal obesity was defined as waist circumference of more than 90 cm for men and more than 85 cm for women. Smoking was categorized as current, ex-smoker of less than 1 year, or non-smoker, according to smoking status. The presence of dyslipidemia was defined by low-density lipoprotein (LDL) ≥ 130 mg/dL in patients with hypertension, and also included cases already receiving lipid-lowering agents [9]. Patients were considered to have diabetes mellitus if their fasting plasma glucose level was ≥126 mg/dL, if their HbA1c level was ≥6.5 %, or if they were taking oral hypoglycemic drugs or insulin.

Target organ damage was evaluated by electrocardiographic LVH for the heart, by estimated glomerular filtration rate (GFR) of <60 mL/min/1.73 m^2^, and/or by an albumin to creatinine ratio of ≥30 μg/mg. Renal impairment was defined as having an estimated GFR < 60 mL/min, as calculated by the Modification of Diet in Renal Disease equation. Microalbuminuria was defined as an albumin to creatinine ratio of 30 to 300 μg/mg [10]. Electrocardiographic LVH was identified if the result of the QRS duration multiplied by the Cornell voltage combination (R in aVL + S in V3, with 8 mm added in women) was higher than 2440 mVms [11], or if Sokolow-Lyon voltage (S in V1 + R in V5/6) was higher than 38 mm [12].

Cardiovascular disease included coronary artery disease, congestive heart failure, and stroke. The presence of coronary artery disease was defined as acute myocardial infarction or hospitalization with angina pectoris. The presence of congestive heart failure was defined as the need for hospitalization. Diagnosis of stroke was necessary to confirm infarction demonstrated by computed tomography or magnetic resonance imaging.

### Statistical analysis

All statistical analyses were performed using SPSS version 18.0 (SPSS Inc., Chicago, IL, USA). Interval variables were described as mean ± standard deviation. Nominal or ordinal variables were described as proportions. Student’s *t*-test or one way-ANOVA were used for continuous variables and chi-square test or Fisher exact test were used for the categorical variables. Multivariate logistic regression analysis was performed to determine independent clinical predictors for RH in the stepwise forward selection procedure. All analyses were performed with 95 % confidence intervals, and p-values of less than 0.05 were considered statistically significant.

## Results

**Fig. 1:**
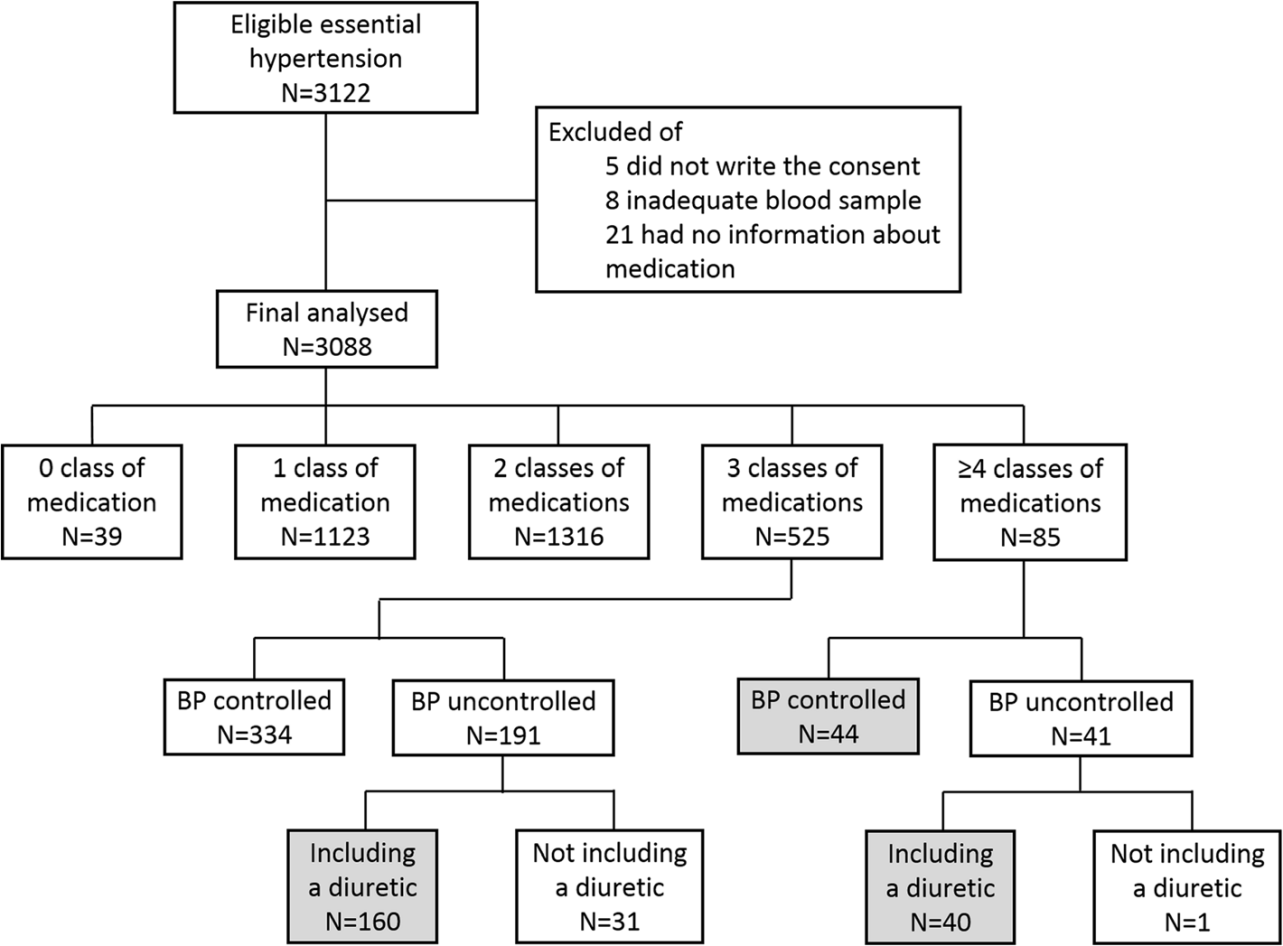
Flow chart of the study: evaluation for confirmation of resistant hypertension. Among 3122 eligible patients, 3088 were enrolled in the study. These patients included 200 with uncontrolled hypertension who were taking three or more classes of antihypertensive medications, including a diuretic, and 44 with controlled hypertension who were taking four or more antihypertensive medications

Among 3122 recruited patients, 34 patients were excluded: 5 did not sign an informed consent form, 8 had inadequate blood samples, and 21 provided no information about antihypertensive medication (Fig. 1). Therefore, 3088 patients were included in the analysis.

### Baseline characteristics

The prevalence of RH among all hypertensive patients was 7.9 % (*N* = 244). The mean age of the patients was 64.3 ± 11.3 years (Table 1) and 48.3 % of patients were men. The average systolic blood pressure and diastolic blood pressure of patients were 131.7 ± 15.2 mmHg and 79.4 ± 10.0 mmHg, respectively. On average, patients were taking 1.84 ± 0.82 classes of medication, primarily angiotensin-converting-enzyme (ACE) inhibitors or angiotensin-II receptor blockers (ARBs, 70.0 %), followed by calcium channel blockers (CCB, 56.5 %). In comparison to those with non-RH, patients with RH were more likely to be men and to have significantly higher BMI and increased blood levels of fasting plasma glucose and HbA1c.

**Table 1:** Baseline characteristics of patients with resistant and nonresistant hypertension

Characteristics	Resistant (*N* = 244)	Nonresistant (*N* = 2844)	*P*-value	Total (*N* = 3088)
Age, yr	64.7 ± 12.7	64.3 ± 11.2	0.621	64.3 ± 11.3
Male, *n* (%)	135 (55.3)	1355 (47.6)	0.021	1490 (48.3)
Average systolic BP, mmHg	146.6 ± 15.6	130.4 ± 14.5	<0.001	131.7 ± 15.2
Average diastolic BP, mmHg	83.9 ± 11.4	79.0 ± 9.7	<0.001	79.4 ± 10.0
Waist, cm	91.4 ± 9.2	88.3 ± 8.9	<0.001	88.5 ± 9.0
Abdominal obesity, *n* (%)	163 (66.8)	1584 (55.7)	0.001	1747 (56.6)
Body mass index, kg/m^2^	26.2 ± 3.3	25.0 ± 3.1	<0.001	25.1 ± 3.2
Smoking			0.004^c^	
Current smoker, *n* (%)	62 (25.4)	491 (17.3)	0.001^b^	553 (17.9)
Ex-smoker, *n* (%)	58 (23.8)	668 (23.5)		726 (23.5)
None, *n* (%)	124 (50.8)	1685 (59.2)		1809 (58.6)
Diabetes and prediabetes			0.046^c^	
Diabetes mellitus, *n* (%)	86 (35.2)	825 (29.0)	0.04^b^	911 (29.5)
Impaired fasting glucose, *n* (%)	112 (45.9)	1391 (48.9)		1503 (48.7)
Non-diabetes, *n* (%)	46 (18.9)	628 (22.1)		674 (21.8)
Fasting plasma glucose, mg/dL	112.2 ± 2.4	106.9 ± 0.7	0.031	107.3 ± 36.8
HbA1c, %	6.31 ± 1.06	6.15 ± 0.95	0.017	6.16 ± 0.96
Serum creatinine, mg/dL	1.09 ± 0.26	1.03 ± 0.25	<0.001	1.03 ± 0.25
Estimated GFR, mL/min/1.73 m^2^	66.6 ± 17.5	69.3 ± 15.7	0.02	69.1 ± 15.8
< 60, *n* (%)	77 (32.1)	621 (22.2)	<0.001	698 (23.0)
Albumin:creatinine ratio, mg/g	82.0 ± 424.2	53.2 ± 240.8	0.296	55.5 ± 260.0
< 30, *n* (%)	177 (72.5)	2179 (76.6)		2356 (76.3)
30–300, *n* (%)	56 (23.0)	567 (19.9)		623 (20.2)
> 300, *n* (%)	11 (4.5)	98 (3.4)		109 (3.5)
LVH on ECG, *n* (%)	21 (8.7)	110 (3.9)	<0.001	131 (4.2) ^c^
Lipid profiles				
Total cholesterol, mg/dL	182.5 ± 36.7	183.1 ± 36.2	0.812	183.0 ± 36.2
LDL, mg/dL	103.1 ± 33.5	105.7 ± 32.5	0.243	105.5 ± 32.6
HDL, mg/dL	44.9 ± 11.1	46.9 ± 12.2	0.014	46.7 ± 12.2
Triglyceride, mg/dL	191.6 ± 144.1	162.3 ± 100.1	0.002	164.6 ± 104.5
Dyslipidemia, *n* (%)	48 (19.7)	645 (22.7)	0.28	693 (22.4)
Total number of classes of medications	3.34 ± 0.48	1.71 ± 0.70	<0.000	1.84 ± 0.82
Baseline antihypertensive medications				
Diuretics, *n* (%)	242 (99.2)	907 (31.9)	<0.001	1149 (37.2)
Calcium channel blocker, *n* (%)	213 (87.3)	1533 (53.9)	<0.001	1746 (56.5)
ACE inhibitor/ARB, *n* (%)	237 (97.1)	1926 (67.7)	<0.001	2163 (70.0)
β-blocker, *n* (%)	118 (48.4)	480 (16.9)	<0.001	598 (19.2)
α-blocker, *n* (%)	4 (1.6)	8 (0.2)	0.005^a^	10 (0.3)
Central acting drugs, *n* (%)	0 (0)	0 (0)		0 (0)
Direct vasodilator	2 (0.8)	2 (0.1)	0.034^a^	4 (0.1)
Comorbidities				
Stroke, *n* (%)	16 (6.6)	118 (4.1)	0.076	134 (4.3)
Cardiovascular disease, *n* (%)	43 (17.6)	306 (10.8)	0.001	350 (11.3)
Renal disease, *n* (%)	11 (4.5)	121 (4.3)	0.851	132 (4.2)
Peripheral arterial disease, *n* (%)	16 (6.6)	119 (4.2)	0.082	136 (4.4)

*Abbreviations*: *α-blocker* alpha blocker, *ACE inhibitor* angiotensin-converting enzyme inhibitors, *ARB* angiotensin-II receptor blockers, *β-blocker* beta-blocker, *BP* blood pressure, *ECG* electrocardiograph, *GFR* glomerular filtration rate, *HbA1c* glycosylated hemoglobin, *HDL* high-density lipoprotein, *LVH* left ventricular hypertrophy, *LDL* low-density lipoprotein

^a^Fisher’s exact test was used

^b^Rate of the variable was compared with the rest

^c^The results of the one-way ANOVA with Bonferroni’s test for post-hoc comparisons

### Blood pressure and medications

**Fig. 2:**
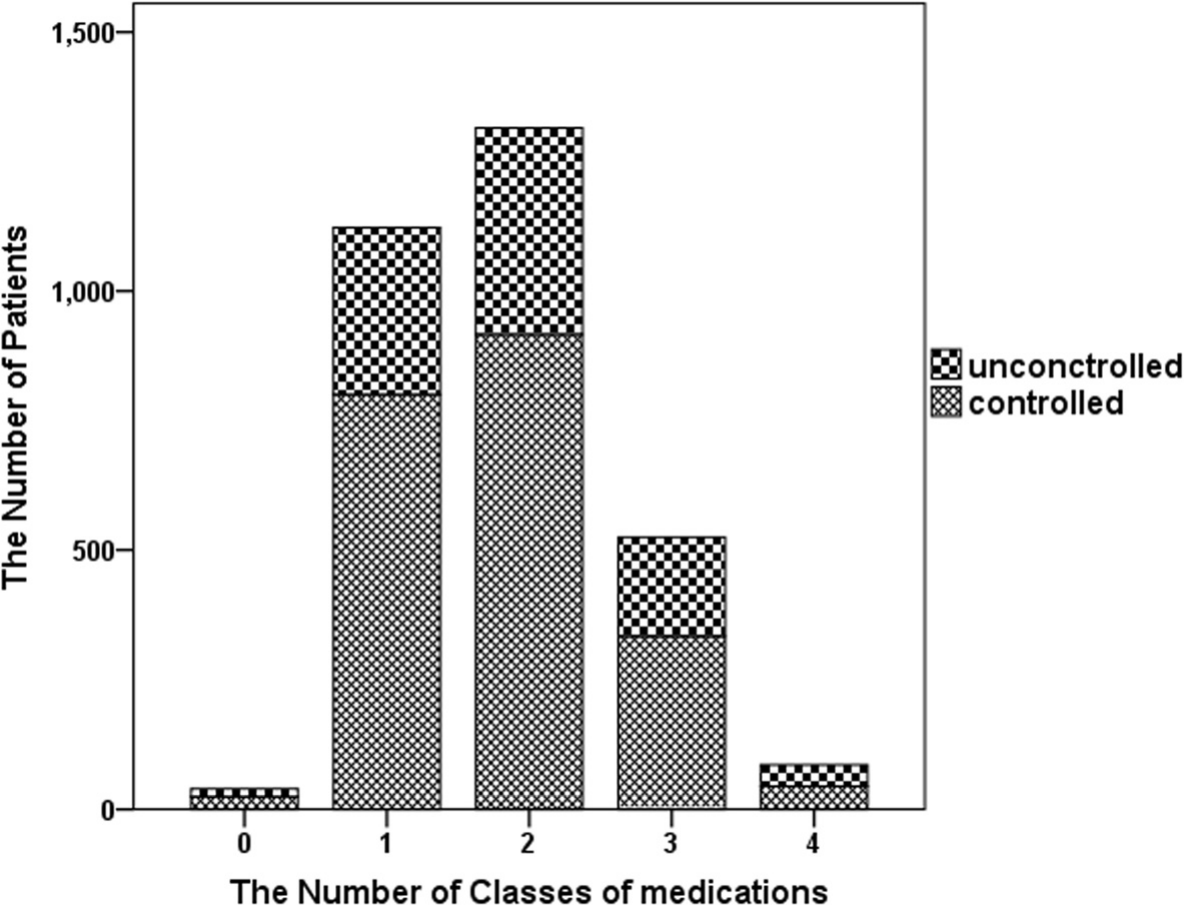
The proportion of patients with controlled and uncontrolled hypertension taking one, two, three, or four or more classes of medication. Among patients with controlled hypertension, 61.5 % (*N* = 24) took no medication, 71.3 % (*N* = 801) took one type of medication, 69.6 % (*N* = 916) took two, 63.6 % (*N* = 334) took three, and 51.8 % (*N* = 44) took four or more

The prevalence of RH in all hypertensive patients was 7.9 % (*N* = 244, grey boxes in Fig. 1), 19.8 % of patients were prescribed three or more classes of antihypertensive medications (*N* = 610). Systolic and diastolic blood pressure were well controlled in 2119 patients (68.6 %). Among patients who were prescribed any medication, 71.3 % (*N* = 801) had controlled blood pressure. The percentage of patients with controlled blood pressure varied according to the number of medications prescribed: one medication, 69.6 % (*N* = 916); two medications, 63.6 % (*N* = 334); three medications; and four or more medications, 51.8 % (*N* = 44, Fig. 2). Among patients with RH, the most commonly prescribed medications were diuretics (99.2 %), ACE inhibitors/ARBs (97.1 %), and calcium channel blockers (87.3 %).

### Cardiovascular risk factors

Compared with patients with non-RH, those with RH had more cardiovascular risk factors at baseline, such as abdominal obesity, smoking, diabetes mellitus, and renal impairment. Significantly more RH patients were current smokers, than were non-RH patients (25.4 and 17.3 %, respectively; *P* = 0.001). Renal impairment, defined as an estimated glomerular filtration rate (GFR) of <60 mL/min/1.73 m^2^, was more prevalent among RH patients as it was among non-RH patients (32.1 % versus 22.2 %, respectively). However, the prevalence of dyslipidemia did not significantly differ between patients with RH and those with non-RH.

### Target organ damage and comorbidities

**Fig. 3:**
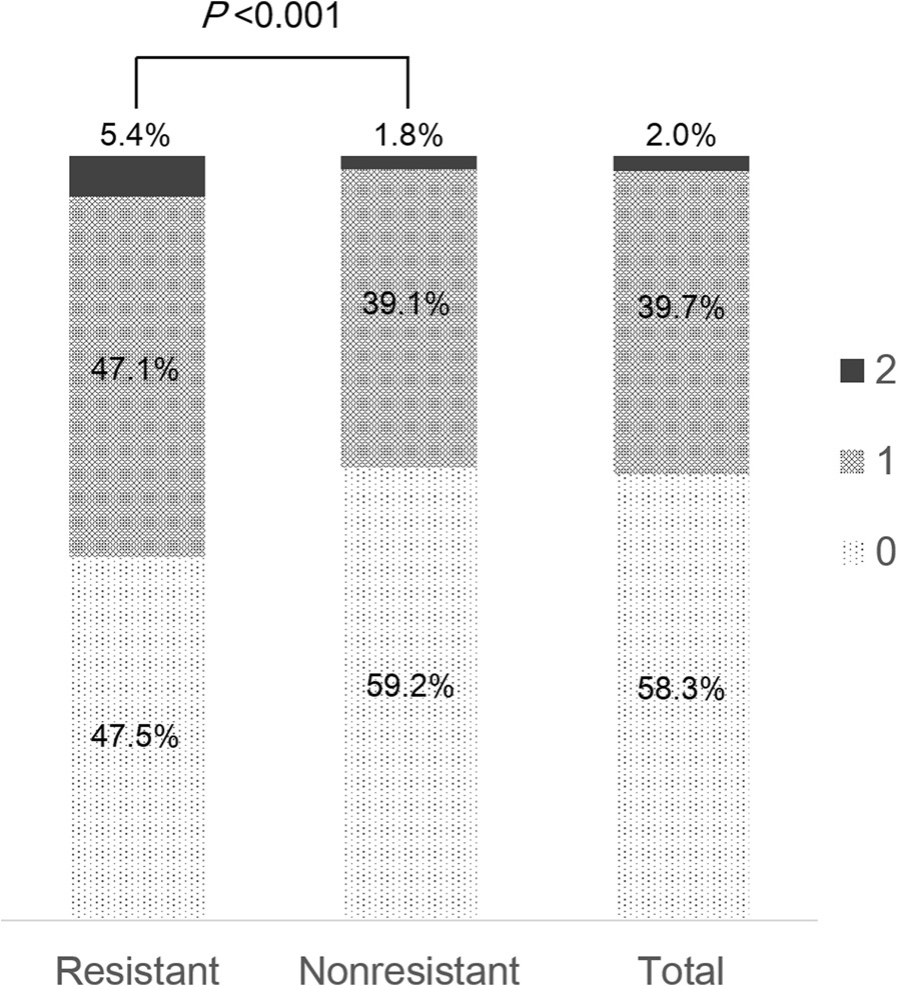
Target organ damage among patients with resistant and nonresistant hypertension. Cardiac and renal target organ damage was more frequently detected in patients with resistant hypertension than in those with nonresistant hypertension (*P* < 0.001)

Target organ damage to the heart and/or the kidney was observed in 1284 patients (41.7 % of total participants, Fig. 3). Target organ damage was more prevalent in RH patients (*P* < 0.001). Among total participants, 131 patients (4.2 %) had cardiac involvement, which was defined by LVH on electrocardiography, and 1217 patients (39.6 %) had renal involvement, which was defined by an estimated GFR < 60 mL/min/1.73 m^2^ or an albumin to creatinine ratio of ≥30 μg/mg (Table 2). Among patients with RH, the prevalence of LVH and decreased estimated GFR were significantly higher (both *P* < 0.001). Although the prevalence of microalbuminuria and/or overt proteinuria were not statistically significant between two groups, these conditions tended to be higher among patients with RH than among those with non-RH.

**Table 2:** Prevalence of hypertensive target organ damage among participants

*n* (%)	Resistant	Nonresistant	*P-*value	Total
Cardiac damage	21 (8.7)	110 (3.9)	<0.001	131 (4.2)
LVH on ECG	21 (8.7)	110 (3.9)	<0.001	131 (4.2)
Renal damage	120 (49.6)	1097 (38.7)	0.001	1217 (39.6)
Estimated GFR <60	77 (32.1)	621 (22.2)	<0.001	698 (23.0)
Albumin:creatinine ratio ≥30	67 (27.8)	665 (23.8)	0.158	732 (24.1)

*Abbreviations*: *ECG* electrocardiograph, *GFR* glomerular filtration rate, *LVH* left ventricular hypertrophy

### Predictors of resistant hypertension

Table 3 shows the results of multivariate logistic regression analysis, which were adjusted for significant variables in a univariate analysis. The significant variables identified as predictors of RH among all hypertensive patients, in increasing order of odds ratio were: electrocardiographic LVH, current smoking, renal impairment (estimated GFR <60 mL/min/1.73 m^2^), abdominal obesity (waist circumference ≥90 in men and ≥85 in women), and the presence of cardiovascular disease. Electrocardiographic LVH predicted the greatest odds of RH (odds ratio [OR] 2.23, 90 % CI 1.34–3.71). Gender, microalbuminuria or macroalbuminuria, and diabetes mellitus did not significantly differ between the groups.

**Table 3:** Predictors of resistant hypertension at primary clinics (*N* = 3088)

Variables	Adjusted Odds Ratio^a^	95 % CI	*P-*value
LVH on ECG	2.23	1.34–3.71	0.002
Current smoker	1.75	1.27–2.40	0.001
Estimated GFR <60	1.65	1.23–2.22	0.001
Abdominal obesity	1.60	1.20–2.13	0.001
Cardiac disease	1.50	1.04–2.17	0.032

*Abbreviations*: *ECG* echocardiograph, *GFR* glomerular filtration rate, *LVH* left ventricular hypertrophy

^a^The result of the binary logistic regression analysis included significant variables for men, abdominal obesity, smoking, diabetes mellitus, electrocardiographic LVH, estimated GFR < 60 mL/min/1.73 m^2^, microalbumuria/macroalbuminuria and cardiovascular disease

## Discussion

This observational and cross-sectional study surveyed demographic findings, clinical characteristics, and antihypertensive medication classes in hypertensive patients at 230 primary care clinics in Korea. The purpose of our study was to evaluate the prevalence of the patients who corresponded to the criteria of RH, and to determine the demographic and clinical features that distinguish high-risk individuals who develop RH from all hypertensive patients.

In this study, the prevalence of RH was 7.9 % (*N* = 244), which is lower than the prevalence reported in some studies. This phenomenon may be explained by the different conditions of our study, as compared with other studies. In the Controlled Onset Verapamil Investigation of Cardiovascular Endpoints (CONVINCE) trial and the Antihypertensive and Lipid Lowering Treatment to Prevent Heart Attack Trial (ALLHAT), the prevalence of RH was 18 and 15 %, respectively. Those data were examined from patients who were 55 years or older who had one or more additional cardiovascular risk factors [13, 14]. In another study, target blood pressure was defined as less than 130/80 mmHg if the patients had diabetes mellitus or kidney disease, which led to 9.1 % of patients defined as having RH [6]. In our study, however, patients were 18 years or older and did not require any cardiovascular risk factor for enrollment. We used the new Joint National Committee 8 guideline that define target blood pressure (<140/90 mmHg) to be the same for patients with and without diabetes mellitus and kidney disease [8]. Another hypothesis, which may help explain the lower prevalence of RH in our study, is that those patients with uncontrolled hypertension may have already been referred to the hospital for further evaluation and clinical management. According to the 2013 Report of Assessment for quality of hypertension treatment in Korea, about 30 % of hypertensive patients receive medications from hospitals, rather than primary clinics [15]. Thus, the prevalence of RH at primary clinics may be lower than that of RH at hospitals.

Moreover, the prevalence of RH may be overestimated at primary clinics. In the present study, the prevalence of uncontrolled hypertension was 31.4 %, much higher than that reported in other studies (13–17 %) [5]. Forced titration of antihypertensive medications by the physician may also contribute to the improved control rate of hypertension. If patients had uncontrolled RH and were taking three medications, including a diuretic, they would be reallocated from the RH group to the controlled non-RH group; thus, the prevalence of RH would be decreased.

We showed several predictors to be associated with RH: electrocardiographic LVH, renal impairment, current smoker, abdominal obesity, and cardiovascular disease. In particular, hypertensive patients with electrocardiographic LVH were 2.3 odds (1.39–3.80, 95 % confidence interval) more likely to have RH compared to those without electrocardiographic LVH. LVH is not only one of the most important subclinical cardiac alterations that result from continuous high blood pressure but also represents a target organ damage [16]. In addition to a chronic pressure overload, overexpression of humoral and hormonal factors are also attributed to the development of cardiac hypertrophy in patients with RH and obstructive sleep apnea, hyperaldosteronism, or both [17]. Although it has yet to be established whether LVH aggravates hypertension, if patients with uncontrolled hypertension have electrocardiographic LVH, further evaluation for the attributed causes of LVH may be needed to improve the clinical management of hypertension.

The prevalence of RH in the subgroup with renal impairment (*N* = 698) was 11.0 %, in our study. Previous studies have reported the prevalence of RH to be 50 % or more [18]. Chronic renal parenchymal disease has been ascertained as the most common identifiable secondary cause of RH. Possible mechanisms, by which decreased renal function leads to development of RH, include the retention of sodium and fluid and the up-regulation of the renin-angiotensin system [19, 20]. Thus, the use of diuretics and ACE inhibitors or ARBs should be considered in patients with increased serum creatinine or estimated GFR.

Lifestyle modifications, such as weight reduction and smoking cessation, may also help improve hypertension management. Although the exact mechanism by which obesity increases blood pressure is not well understood, excess weight gain has been reported as the best predictor for the development of hypertension [21]. Obesity can lead to increased renal sodium reabsorption and renal injury through the activation of the renin-angiotensin system and increased sympathetic tone [22].

Identifying individuals at high risk of developing RH, who have electrocardiographic LVH, renal impairment, abdominal obesity, current smoking, or cardiovascular disease, is important for the selection of appropriate antihypertensive medications. Difficulty in controlling blood pressure in patients with the aforementioned predictors should prompt earlier consideration of forced titration of medications and evaluation for secondary hypertension.

This study has some limitations. First, drug adherence was not measured accurately. Poor adherence to antihypertensive medications is a well-known, major cause of failure to reach target blood pressure [23]. However, pill count and patient education were performed at each visit to the clinic. Second, although we excluded patients with a history of secondary or white-coat hypertension from the initial recruiting, estimation of the prevalence of pure RH was not perfect. It was difficult to perform sophisticated examinations for all patients with uncontrolled hypertension in the primary care setting. The final objective of this study was to manage hypertension effectively by identifying patients who corresponded with the criteria of RH. If secondary or white-coat hypertension is in doubt, referral to a hospital or hypertension-specialized institution may be indicated. However, the results of our study are valuable because they represent the first investigation of RH at primary clinics in Korea, and are robust, in that they are derived from 3088 patients and 247 primary physicians.

## Conclusions

The prevalence of RH at primary clinics in Korea was 7.9 %, which is relatively low when compared with the findings of studies conducted in other countries. Predictors for increased risk of RH were electrocardiographic LVH, renal impairment, current smoking, abdominal obesity, and the presence of cardiovascular disease. Therefore, these predictors may be helpful for detecting risk of RH and for improving the efficiency of clinical management for hypertension by forced titration or selection of drugs.

## Abbreviations

BP: blood pressure
CI: confidence interval
GFR: glomerular filtration rate
LVH: left ventricular hypertrophy
OR: odds ratio
RH: resistant hypertension
TOD: target organ damage
